# Interactions between Layered Double Hydroxide Nanoparticles and Egg Yolk Lecithin Liposome Membranes

**DOI:** 10.3390/molecules28093929

**Published:** 2023-05-06

**Authors:** Bin Liu, Yanlan Wang, Na Du

**Affiliations:** 1 School of Chemistry and Chemical Engineering, Liaocheng University, Liaocheng 252059, China; binliu@lcu.edu.cn; 2 Key Laboratory of Colloid and Interface Chemistry (Ministry of Education), School of Chemistry and Chemical Engineering, Shandong University, Jinan 250100, China

**Keywords:** LDHs, liposomes, interactions, membranes

## Abstract

The burgeoning need to study the applications of nanoparticles (NPs) in biomedical and pharmaceutical fields requires an understanding of their interactions with lipid membranes for further in vivo studies. In this paper, negatively charged egg yolk lecithin liposome (EYL) has been prepared and used as model lipid membranes. Positively charged Mg_3_Al-layered double hydroxides (LDHs) are viewed as models of clay particles. The ability of the LDH NPs, a two-dimensional nanostructure with an average diameter of 100 nm (LDHs-100) or 500 nm (LDHs-500) to cross the membranes, has been thoroughly investigated via (high-resolution) transmission electron microscopy (TEM), optical microscopy (OM), scanning electron microscopy (SEM), confocal fluorescence microscopy (CLSM), and dynamic light scattering (DLS). The liposomes with an average diameter of 1.5 μm were prepared by the thin-film rehydration method followed by an extrusion technique. A calcein leakage assay and steady-state fluorescence measurement displayed the variation of membrane integrity and polarity of the pyrene-located microenvironment during the interaction between EYL and calcein-interacted LDH NPs (CE-LDHs) or LDH NPs, respectively. These results imply that not only spherical particles but also even more sophisticated nanostructured materials are able to effectively cross the lipid bilayers, thereby engineering new compounds that may be encapsulated for safe and potential use in biomedical applications.

## 1. Introduction

Motivated by the burgeoning need to explore the possible applications of nanoparticles (NPs) in various pharmaceutical and biological fields as well as their roles taken in the origin of life, intensive research focused on the interaction of phospholipid membranes with NPs, such as SiO_2_ NPs, Au NPs, TiO_2_, etc., has been conducted over the last few decades. On the one hand, these inorganic/lipid hybrid materials can be used for controlled release, drug delivery, and fundamental studies [1,2,3,4,5,6]. On the other hand, it was found that NPs, especially clay minerals, may play an active role in the abiotic origin of life [7,8,9].

Considering that the phospholipid bilayer is the universal component of all cell membranes, it is, therefore, smart to draw lessons from NP–lipid bilayer interactions to shed light on NP–cell interactions. Mornet and co-workers observed supported lipid bilayers (SLB) on silica NPs through cryo-TEM via subsequent adsorption, deformation, and rupture of liposome on the surfaces of either pristine or functionalized silica NPs carrying low negative, positive, or neutral charges [10]. Gradzielski et al. further produced hybrid colloids composed of high loading of SLB-covered silica NPs contained in phospholipid vesicles via the NP adsorption, engulfing, and internalization processes [11]. Recently, Dasgupta and co-workers prepared flavonoid-loaded human serum albumin NPs and lipid membranes with different charges as membrane models. The authors demonstrated that their interaction largely depends on the sizes of particles and vesicles: SLB could be formed when the former ones are larger; on the contrary, SLB@vesicles are preferred [12]. Although several authors published research studies and established findings [3,13,14,15], it remains a great challenge to thoroughly understand the interactions between NPs with various shapes or sizes and cell membranes, such as two-dimensional nanostructured LDH NPs instead of spherical silica NPs. Their trans-membrane dynamic process is still needed to be further explored.

Layered double hydroxides (LDHs) are a large class of layered compounds [16,17], with the general formula $[M_{1-x}^{2+}M_{x}^{3+}{\left(\mathrm{OH}\right)}_{2}]{\left(A^{n-}\right)}_{x/n}\;{\mathrm{mH}}_{2}O$, where $M^{2+}$ and $M^{3+}$ are the divalent and trivalent cations, respectively, and $A^{n-}$ is the exchangeable interlayer anion. The lamellar structure and anion exchange property of LDHs enable drugs or biomolecules to be intercalated into their interlayers to form drug- or bio-LDH nanohybrids [18,19,20]. LDHs used as drug carriers are advantageous owing to their suitable biocompatibility, low toxicity, and biodegradation [19]. We have found that LDHs can induce vesicle formation in surfactant solutions, yielding nanocomposites of LDHs encapsulated in vesicles from the mixture of two simple amphiphiles [21,22]. The authors further reported that (drug-LDHs)@liposome nanocomposites have excellent water dispersity and enhanced sustained-release performances in comparison with drug–LDH nanohybrids, thereby engineering LDH-based delivery systems for different drugs [23,24,25]. In this work, we mixed prepared liposomes with LDH NPs instead of creating an LDHs@liposomes hybrid by adding lipid molecules with LDH NPs simultaneously in order to really follow the trans-membrane process of drugs@LDHs, which are apparently needed in order to realize the possibility for drugs@LDHs to be further used in vivo studies. Inspired by the pioneering works and reflecting the works we have performed, we employ synthesized LDHs, an anionic clay (the ζ potential of the LDH NPs was 29.5 ± 0.5 mV, 0.5 g/L), and a two-dimensional nanostructured inorganic material, as a model of clay particles and egg yolk lecithin liposome (EYL, −36.8 ± 1.5 mV, 10 g/L, and pH = 8) that are close to 1.5 μm. We studied well-defined mixtures of EYL liposomes and LDH particles in an aqueous solution (pH = 8). The interaction between LDHs and EYL is investigated, with the particular aim to quantitatively study when and how the transport of LDH NPs through the EYL membrane and into its interior proceeds and what hybrid nanostructures are formed during this process, and the possible trans-membrane mechanism of LDH particles is also proposed. Supported membranes could be formed on the anionic clay surfaces of LDH NPs with varying sizes and then fused with other liposomes, confirming the possible value of LDH NPs on present-day cellular membranes. In other words, these results may help to understand its possible contributions to the modern origin of life.

## 2. Results and Discussion

### 2.1. The Formation of EYL

**Figure 1:**
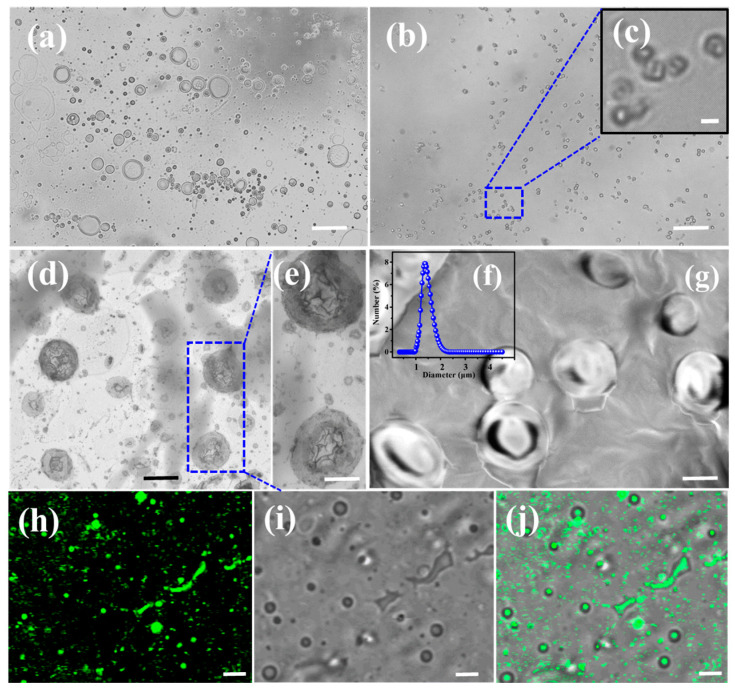
Optical microscopy images of liposomes prepared (**a**) without using and (**b**,**c**) using extruder with two stacked polycarbonate membranes (pore size = 3 μm), (**d**,**e**) negative-staining TEM image of liposomes after extrusion process, (**f**) DLS size distributions of liposomes after extrusion, (**g**) SEM image of liposomes after extrusion process, and (**h**–**j**) CLSM image of CE@EYL: (**h**) bright filed, (**i**) dark filed, and (**j**) merged image. Scale bar: (**a**) 25 μm (**b**) 10 μm, (**d**,**e**,**g**) 2 μm, and (**c**,**h**–**j**) 5 μm.

A majority of unilamellar EYL is observed at the yolk lecithin concentration of 10 g/L using an optical microscope (Figure 1a), of which the size distribution peak decreases from around 3.5 μm to 1.5 μm (Figure 1b–g and Figure S1a,b), revealed by negative-staining TEM and SEM after extruding 11 times with a polycarbonate membrane liposome extruder with a loaded pore size of 3 μm. Meanwhile, the DLS results show that the hydrodynamic diameter of these aggregates is located at 1.5 μm (Figure 1f), which is in agreement with the results from microscopy observation. The appearance of the yolk lecithin solution turns from milky white and opaque (inset photo in Figure S1a) to translucent (inset photo in Figure S1b) after using extruders, indicating the decreasing size of EYL. To further confirm the existence of the core–shell structure in the aggregates, the hydrophilic fluorescence of calcein was used to mark the water cores of liposomes. Green light-emitting spherical spots are observed for the calcein-marked solution, thereby confirming the existence of inner water cores in the aggregates (Figure 1h,i).

### 2.2. The Formation of LHDs and Calcein-Loaded LDHs

**Figure 2:**
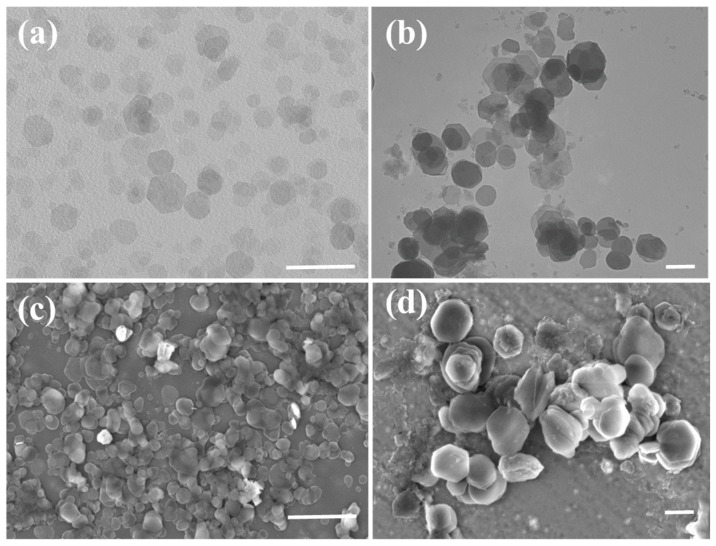
(**a**,**b**) Negative-staining TEM image of LDHs-100 and LDHs-500; (**c**,**d**) SEM images of 100 nm and 500 nm LDH NPs. Scale bar: 500 nm.

Pristine LDHs-100, LDHs-500, and calcein-loaded LDHs-100 (CE-LDHs-100) were used to check, by electron and fluorescence microscopy, the cellular uptake pathway of the LDHs. The TEM and SEM results indicate that all the LDH particles have characteristic hexagonal plate-like morphology with uniform size (Figure 2). Meanwhile, the DLS results show that the hydrodynamic diameters of LDHs-100 and LDHs-500 are located at 110 nm and 515 nm, which is in agreement with the TEM observation Figure S1b,c. The powder X-ray diffraction patterns showed that each LDH had a typical LDH structure, and the *d*-spacing of each LDH determined was 0.89, 0.79, and 1.60, respectively (Figure S2 and Table S1), upon the molecular size of anions such as NO_3_^−^, Cl^−^, and FITC, as previously reported [26,27]. The position of the (003) diffraction peak in the CE-LDHs-100 (Figure S3c) SAXS spectrum shifted significantly compared to LDHs-100, indicating that the calcein molecules replaced the nitrate anions into the LDHs-100 layers, and the interlayer distance increased from 0.89 nm to 1.60 nm (Table S1). There is a good multiple relationship between the (003), (006), and (009) diffraction peaks of the samples, indicating that the prepared sample crystal structure is good. After the intercalation, the position of the (110) diffraction peak, which represents the density of the atoms on the laminate, did not change significantly, indicating that the ion exchange process did not destroy the laminate structure.

The results of the analysis of the calculation of the sample unit cell parameters are present in Table S2, where *a* represents the atomic arrangement density on the laminate, *b* represents the metal ion spacing, and *c* reflects the unit cell thickness. There was little change in the size of *a,* indicating that there was no change in the type and proportion of atoms on the plate after calcein intercalation. The *c*-value increased from 2.67 nm to 4.80 nm. The *c* value reflects the charge density of the laminate, the type of anions between the layers, and their arrangement, and the increase in the *c* value is caused by the significant increase in the spacing between the CE-LDHs-100 layers.

In the calcein infrared spectrogram (Figure S4a), the C-H telescopic vibration absorption peaks on the non-benzene ring are 3003 cm^−1^ and 2807 cm^−1^. The peak of 1627 cm^−1^ belongs to the C=C double bond vibration absorption peak on the non-benzene ring. The four peaks of 1601 cm^−1^, 1546 cm^−1^, 1509 cm^−1^, and 1444 cm^−1^ belong to the vibration absorption peak of the benzene ring C=C double bond skeleton; 1391 cm^−1^ belongs to the absorption peak produced by C-H bending vibration; 1287 cm^−1^ and 1253 cm^−1^ is C-N telescopic vibration; 1209 cm^−1^ and 1177 cm^−1^ is the characteristic absorption peak of C-C(=O)-O; 851 cm^−1^, 760 cm^−1^, and 696 cm^−1^ is C-H and O-H external bending vibration.

In the LDHs-100 infrared spectrogram (Figure S4b), the strong and wide absorption peak at about 3451 cm^−1^ is made up of two or three hydroxyl telescopic vibrations and the elastic vibration of interlayer water molecules, the absorption peak at 1639 cm^−1^ is the plane bending vibration of water molecules, the interlayer anions are nitrate, the absorption peak at 1379 cm^−1^ is the vibration band belonging to NO_3_^−^, and the peak type is sharp. The absorption peaks at 660 cm^−1^ are the telescopic vibration absorption peaks belonging to the Mg-OH bond, and the absorption peaks at 550 cm^−1^ and 469 cm^−1^ are lattice vibration absorption peaks belonging to Mg-O.

In the CE-LDHs-100 infrared spectrogram (Figure S4c), 1588 cm^−1^ and 1451 cm^−1^ are the absorption peak generated by the skeleton vibration on the benzene ring, 1334 cm^−1^ is the telescopic vibration absorption peak of the C-O bond, and 1154 cm^−1^ and 1061 cm^−1^ belong to the C-O telescopic vibration absorption peak on the carboxyl group, moving towards the low wavenumber, which is due to the interaction between the calcein intercalation layer and the LDH layer.

### 2.3. The Permeability of Calcein and Nile Red to the Protocell Membranes

**Figure 3:**
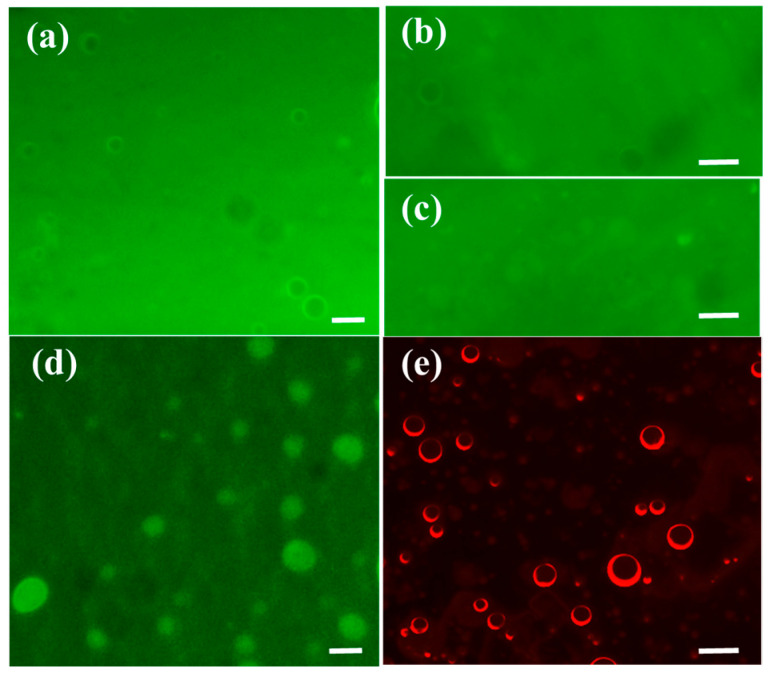
Fluorescence microscope images showing EYL membranes encapsulating calcein into the lumen of liposomes: (**a**) 5 h, (**b**,**c**) 7 days, and (**d**) 10 days after the addition of calcein into EYL solution, and (**e**) Nile red into the hydrophobic domain within the bilayer membranes after 2 h. All scale bar: 5 μm.

The fluorescence microscopic images show that the green color inside the water core is still too weak to distinguish (Figure 3a) after the addition of calcein inside the EYL solution for 5 h, indicating that calcein is not able to cross a majority of liposome membranes in such a short time. A week later, we observe that the fluorescence intensity is increased, implying that a number of negatively charged molecules is passively and gradually taken into the lumen, which is enough to color the lumen of EYL (Figure 3b–d). In other words, compared with vesicle membranes formed by simple surfactants [28], it is difficult for the calcein to cross the EYL membrane owing to the electronic repulsive force between them. By contrast, the oleophilic dye Nile red could cross the membranes and be encapsulated between the hydrophobic shell within 2 h (Figure 3e).

### 2.4. The Interaction between LDHs Particles and Liposome Membranes

**Figure 4:**
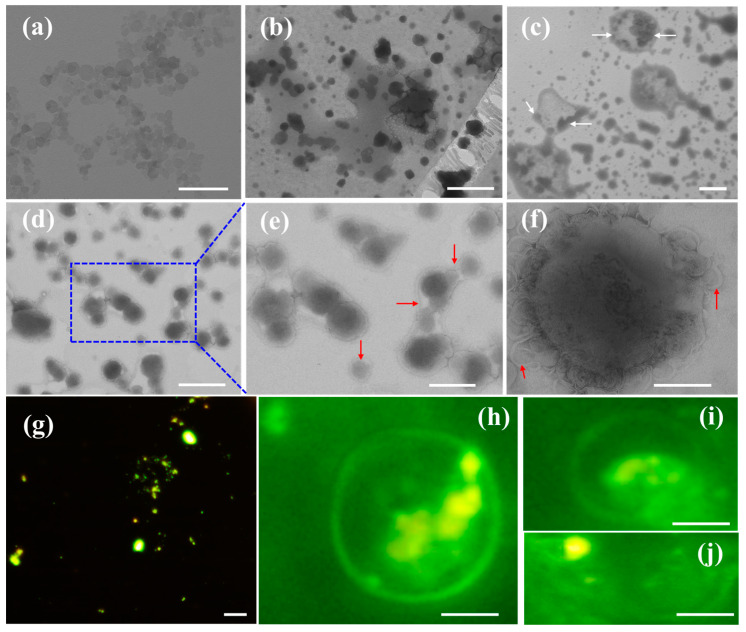
(**a**–**f**) Negative-staining TEM images showing the time-dependent interaction of LDHs-100/EYL (*v*/*v* = 1/4) after standing 30 min, 1 h, 2 h, and 3 days, respectively. (**g**) Fluorescence microscopy images of CE-LDHs-100 and (**h**–**j**) CE-LDHs-100/EYL mixed 2 h before measurement (*v*/*v* = 1:4). The red arrows indicate the outer lipid layer covering the LDHs particle surface, and the white arrows indicate the LDHs particle encapsulated in liposomes. Scale bar: (**a**–**d**,**f**) 1 μm, (**e**) 500 nm, and (**g**–**j**) 2 μm.

The ζ potential of LDHs-100 NPs of 0.5 g/L and EYL of 10 g/L (in bicine buffer, pH = 8, after extruding 11 times) are 29.5 ± 0.5 mV and −36.8 ± 1.5 mV, respectively, which confirms the positively charged LDHs and negatively charged EYL, respectively. LDHs-100 was added to the lipid solution to form the LDHs-100/EYL mixture. It found that the mixture looked white and turbid to the naked eye. Trans-membrane kinetics of LDH NPs were quantitatively recorded by collecting and analyzing samples at different incubation times after mixing EYL and LDH NPs. LDH particles with hexagonal plate-like morphology were observed under NS-TEM observation in the mixture within 30 min (Figure 4a); 1 h later, the LDH particles appeared surrounded by a ring of electron-dense material and LDH particles with fuzzy edges were observed with NS-TEM (Figure 4b), which corresponds to the outer lipid supported bilayer covering the particle surface (LDHs-SLB); 2 h later, as shown by the red arrows in Figure 4e, the two concentric rings correspond to the polar headgroups from the two lipid layers, which scatter electrons more strongly than the hydrocarbon chains. These phenomena arise from the adsorption of lipid and bilayer patches around LDH surfaces, indicating the bilayer formed around the LDH particles; Additionally, as indicated by the red arrows in Figure 4e, there still exists lipid layers outside the bilayer encapsulated LDHs, which were most likely formed by the fusion process. Moreover, an interesting phenomenon was found where LDH particles were encapsulated within the water core of liposomes after 2 h, as indicated by the white arrows in Figure 4c. The size of encapsulated objects in the vesicle is 100 nm, consistent with the scale of LDHs-100 sheets. In addition, the detailed electron microscopy analysis indicates that there are buddings in the surface of LDHs@EYL, which might have been functional in the evolution of biological systems, such as the self-reproduction process (Figure 4f). After the surface-layered structure of LDHs-100 comes into contact with the liposome structure, it is very likely that fusion occurs through the rapid exchange of lecithin molecules between the two layers to form a budding structure [29,30]. These results simply quantified the dynamic membrane changes as well as the kinetics of LDH NP internalization over time, which is in overall agreement with the existing reports on the dynamics of nanoparticles across liposome membranes spanning from a few minutes to several weeks from the aspect of time [10,11]; for instance, continuous nano-supported lipid bilayers and internalized silica NPs could be obtained after 5 min and three weeks incubation, respectively.

In order to corroborate the internalization process of the LDHs, fluorescence microscopy is introduced to our experiments (Figure 4g–j). Green light-emitting spherical spots are observed in the calcein-marked solution, thereby confirming the existence of CE-LDHs-100 (Figure 4g). It found that some green spots could cross the membranes and partially be entrapped in liposomes in 2 h, which results in the deformed surfaces of liposomes (Figure 4i,j). Compared with the TEM technique, fluorescence microscopy is a more convincing tool in this work for making quantitative measurements in the internalization process of LDH NPs by avoiding the occurrence of the drying process.

To better understand the trans-membrane behavior of LDHs-100, the effect of the LDHs-100 trans-membrane process on liposome membrane integrity was investigated using fluorescence emission spectroscopy experiments. The liposome dispersion system (CE@EYL) coated with calcein is mixed with 1 g/L LDHs-100 at a volume ratio of 4:1, and the calcein or Nile red fluorescence emission intensity slowly increases over time (Figure S5). After 5 h, it was sonicated, and the fluorescence intensity was found to increase rapidly, indicating the rapid release of the dye from the liposomes. This experiment shows that the LDHs-100 NP trans-membrane process does not destroy the liposome membrane or form a pore structure that causes the release of dye molecules [10,11,31,32].

**Figure 5:**
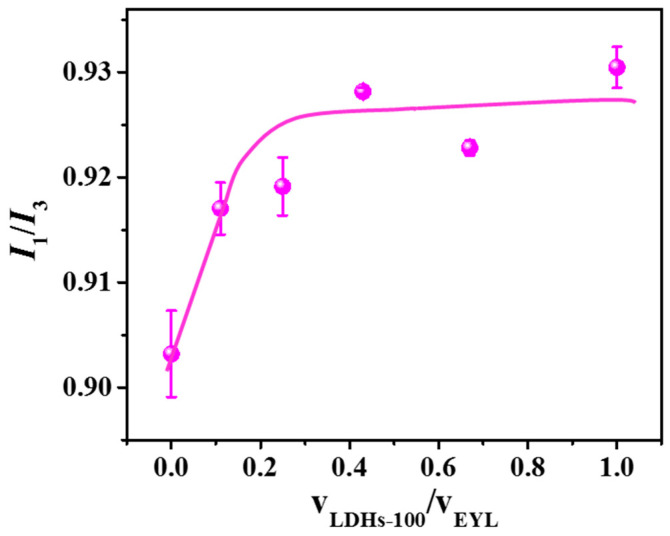
Changes in the fluorescence intensity ratio of pyrene (*I*_1_/*I*_3_) as a function of increasing concentration of LDHs-100.

In addition, pyrene is selected as a probe to study the polarity variation in the bilayer membranes with the increasing addition of LDH NPs. It is reported that the intensity ratio (*I*_1_/*I*_3_) of pyrene in membrane medium, that is, the polarity of the microenvironment, depends on the probe concentration and the polarity of the area; that is, the more pyrene dissolved in the non-polarity area, the lower the value of *I*_1_/*I*_3_, and vice versa [33,34]. We assume that the looser membranes could be formed during the trans-membrane process at the cost of the non-polarity area, which leads to fewer accommodations for probes. In this work, the intensity ratio (*I*_1_/*I*_3_) of pyrene increases slightly with the increasing value of *V*_LDHs-100_/*V*_EYL_ (Figure 5) until it increases to 0.2, thanks to the saturation of LDHs-100 in liposomes. These results confirm our assumptions that the addition of LDHs-100 replaced the nonpolar areas, which results in a higher polarity of the microenvironment.

**Figure 6:**
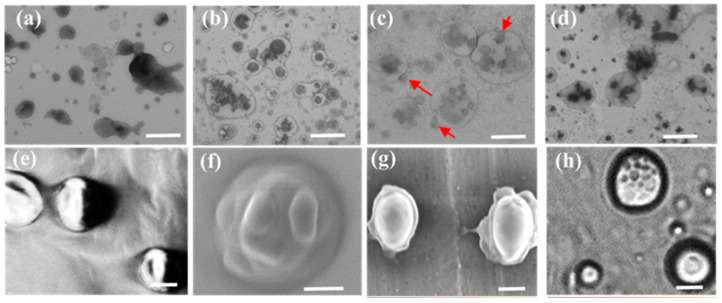
Negative-staining TEM image of LDHs-500/EYL after standing (**a**) 1 h, (**b**) 2 h, (**c**) 4 h, and (**d**) 8 h; (**e**) SEM image of EYL and (**f**,**g**) SEM image of LDHs-500/EYL after standing 2 h; and (**h**) optical microscopy images of LDHs-500@EYL. The red arrows indicate the LHDs-100 covered with lipid layers in the course of fast exchange of lipids with EYL. Scale bar = 2 μm.

In order to investigate the effects of particle size on LDHs-membrane interactions, LDHs with a diameter of 500 nm were synthesized and marked as LDHs-500. A semi-dynamic process of the cellular uptake of LDHs is observed by TEM, SEM, and OM. Firstly, similar to the interaction between LDHs-100 and liposomes, it seems that a great amount of negatively charged lipids adsorbed on the surface of positively charged LDHs-500 (Figure 6a), meaning that LDHs-SLB is formed within 1 h. The fusion process subsequently took place due to electrostatic attractions. The red arrows in Figure 6c indicate the LHDs-100 covered with lipid layers in the course of the fast exchange of lipids with EYL (Figure 6b–d). The SEM images showed that liposome morphology was deformed due to ultra-high vacuum and the lack of water within the SEM chamber (Figure 6e). Figure 6f,g shows us the interaction of LDHs-SLB with liposomes. The optical microscopy images showed that the LDH particles have characteristic hexagonal plate-like morphology with uniform size and are entrapped inside the water core of liposomes (Figure 6h).

HR-TEM was employed to provide further insight into the subtle structures of the composite (Figure S6). It can be seen that there were some sheets encapsulated in the liposome (Figure S6b). The crystal lattice fringes can be clearly seen in LDHs-500@EYL composite and pure LDHs-500, respectively. The distance between the two lattice fringes of encapsulated substance is 0.297 nm, which is close to the crystal lattice space of LDHs-500 sheets of 0.291 nm (Figure S6a). Therefore, this finding confirms that LDHs-500 were encapsulated within the water core of liposomes.

Based on previous reports, it can be expected that the LDH sheets are around 10 nm thick [23]. Dimples are observed in the center of deformed liposomes with an average depth of 110 nm (Figure S7a,c). It is probably due to the collapse of liposomes during the drying process. The sectional analysis results reveal that the LDHs-500@EYL composite is 135 nm thick (Figure S7d), which indicates that the LDH NPs were encapsulated by liposomes.

All the above results illustrate that both LDHs-100 and LDHs-500 are able to effectively cross the lipid bilayer in the same manner, thus allowing us to design and develop new hybrid compounds within a certain average size of LDHs (100 nm ≤ size ≤ 500 nm) that may be encapsulated for safe and potential use in biomedical applications.

### 2.5. The Stability of the LDHs-100@EYL Dispersion

**Figure 7:**
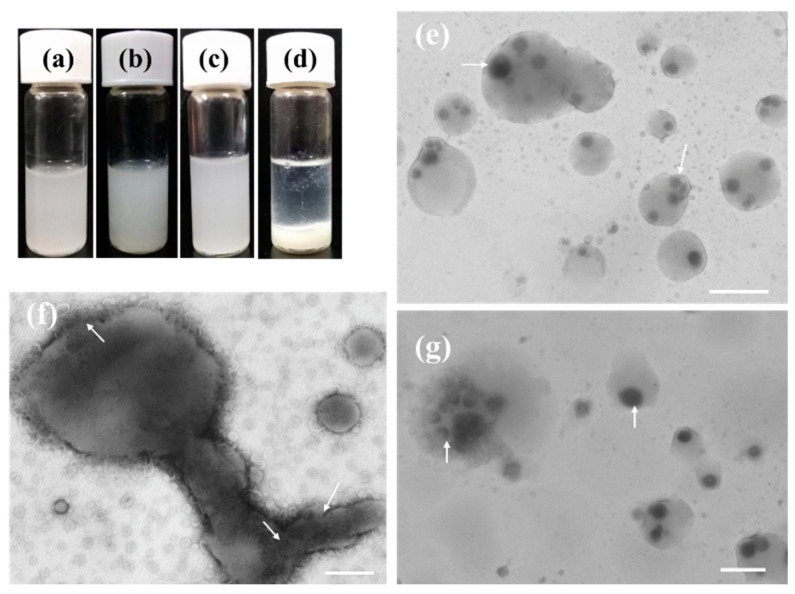
Aqueous dispersions of (**a**,**b**) LDHs-100/EYL after standing 2 h and 2 months, respectively, and (**c**,**d**) LDHs-500/EYL after standing 2 h and 2 months, respectively; (**e**–**g**) NS-TEM images of LDHs-100/EYL after standing 2 months. The white arrows indicate the LDH particle encapsulated in liposomes. Scale bar: (**e**,**g**) 1 μm, (**f**) 500 nm.

Figure 7a–d shows time-dependent images of dispersions of EYL prepared with LDHs-100 (Figure 7a,b) and LDHs-500 (Figure 7c,d) NPs. It is observed that the dispersion of the LDHs-100@EYL system was milky and remained stable after depositing for 2 months at 25 °C even though the fusion process took place between liposomes (Figure 7b,e–g). However, white cloudy precipitation was observed in the aqueous dispersion of LDHs-500/EYL due to the sedimentation of a relatively larger size of LDHs-500 (Figure 7d). LDHs-100@EYL hybrid structures (Figure 7e–g) are observed from the TEM images by collecting aqueous dispersions from Figure 7b. Elongated fused vesicles with budding structures on their surfaces further confirm the occurrence of the LDH NP trans-membrane process (Figure 7f). The formation of bilayers on LDH NPs alters their surface charge and effectively avoids the relative effect of NP–NP repulsion due to the positive charge of LDH NPs, and gravity, which favors setting. Similar results were obtained by others for the dispersion stability of nanoparticles and liposomes [35,36].

### 2.6. The Possible Trans-Membrane Mechanism of LDHs Particles

**Figure 8:**
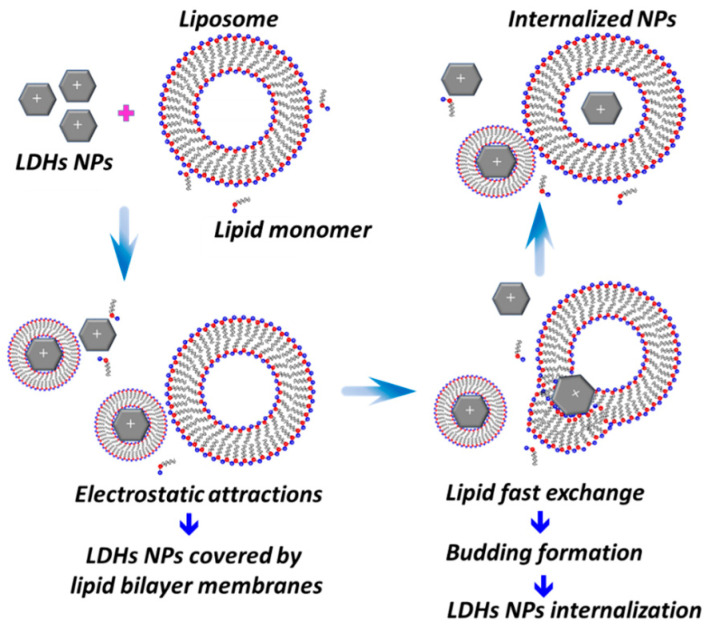
Schematic drawings of the possible mechanisms occurring in the mixed LDHs/EYL systems.

Based on the above discussion, we propose a possible mechanism for the interaction between LDHs and EYL. Similar to the previously reported trans-membrane mechanism of silica NPs and liposomes [1,11,37,38], we propose the mechanism of liposome–LDH interaction as shown in Figure 8. The electropositive LDH surface attracts negatively charged free lipid molecules which facilitates their aggregation into the lipid bilayer. This results in the formation of the lecithin bilayer structure that, once formed, undergoes collision with the liposome bilayer membrane resulting in the rapid exchange of phospholipid molecules that fuse the two to form a budding structure. Eventually, LDH particles cross the liposome bilayer membrane into the water core, completing the trans-membrane process of LDHs. Notably, there is no size effect for the internalization dynamics and morphology evolution route for LDHs-100 and LDHs-500, except that the time stability for as-obtained LDHs-100@EYL is better than LDHs-500@EYL.

## 3. Materials and Methods

### 3.1. Materials

All materials were used without further purification. Egg yolk lecithin (95% purity) and cholesterol (95% purity) were purchased from Heowns Biochem Technologies, Llc., Tianjin, China. Nile red was purchased from TCI. The fluorescence probes 1,6-diphenyl-1, 3,5-hexatriene (DPH), calcein, and pyrene were purchased from Aladdin Industrial Co., Shanghai, China. Magnesium nitrate hexahydrate (99% purity), magnesium chloride hexahydrate (99% purity), aluminum chloride nonahydrate (99% purity), aluminum nitrate nonahydrate (99% purity), and ammonia solution (~25 wt%) were obtained from Sinopharm Chemical Reagent Co., Ltd., Shanghai, China. All other chemicals used were of analytical grade. Water was purified with a Hitech-Kflow water purification system (Hitech, Shanghai, China).

### 3.2. Preparation of Calcein-Loaded Liposomes (Denoted as CE@EYL)

To encapsulate calcein, the dried lipids above were hydrated with 20 mM disodium calcein solution with occasional vibration to disperse the lipid. The sample was then extruded 11 times using 3 μm pore-sized membrane. The resulting solution was then dialyzed for 72 h by using a dialysis bag to remove the unencapsulated free dye. Then, the emission spectra of the dialyzed solution, along with an absorbance-normalized aqueous solution of free dye, were recorded on an LS-55 fluorescence spectrometer (Perkin-Elmer, USA) over a wavelength (λ_em_) range of 470–600 nm by excitation at a wavelength (λ_ex_) of 450 nm. In this experiment, we deducted the effect of dialysis on the concentration of EYL.

### 3.3. LDHs Nanoparticle Synthesis

To prepare small LDH nanoparticles (diameter ≈ 100 nm, denoted as LDHs-100), well-dispersed LDH nanoparticles were synthesized according to the method published by Hou et al. [26]. Briefly, a mixed salt solution of Mg(NO_3_)_2_∙6H_2_O and Al(NO_3_)_3_∙9H_2_O was prepared with an Mg/Al molar ratio of 3.0 and a total salt concentration of 0.5 mol∙L^−1^. The mixed salt solution and an ammonia solution (~6 wt%) were simultaneously added into a beaker under magnetic stirring and N_2_ protection. During this process, the pH value of the reaction system was kept at ~9.5 by altering the relative addition rate of the two raw material solutions. The obtained product was filtered and rinsed with ultrapure water to remove the excess ammonia solution. In the final step, the filter cake was peptized at about 80 °C in oven for about 24 h to convert it into LDHs sol. The recovered white powder was ground in the agate mortar before use.

To prepare even larger LDH nanoparticles (diameter ≈ 500 nm, denoted as LDHs-500), calcination method was used [39]. Briefly, an aqueous stock solution of NaOH, magnesium chloride (2 M), and aluminum chloride were mixed together at the molar Mg/Al/OH ratio of 3:1:10 with magnetic stirring at room temperature. An amount of 12.2 g MgCl_2_∙6H_2_O and 4.83 g AlCl_3_∙9H_2_O mixed in the crucible were calcined for 30 min at 250 °C. A total of 30 mL (30 wt%, 8 g) NaOH solution was added drop-wise to the salt solution under vigorous stirring. Then, the homogeneous solution was transferred into a Teflon-lined autoclave (Taiatsu Glass Ind. Co., Shanghai, China) and heated at 160 °C for 16 h. The sample was dried by storing it in a vacuum oven overnight at 80 °C temperature. Finally, the recovered white powder was ground in the agate mortar before use.

### 3.4. Intercalation of Calcein into LDHs-100 (Denoted as CE-LDHs-100)

For calcein–LDH, a 0.064 M solution of calcein was prepared with (50 mL) of decarbonated water and (0.1 mol/L) NaOH titration until completely dissolved. LDHs-100 particles (1 g) dispersed in decarbonated water (50 mL) were kept under a N_2_ atmosphere. The homogeneous solution was heated at 90 °C for 30 min, and then calcein solution was added to the LDH solution at 120 °C for 20 h. Calcein–LDH was thoroughly washed with decarbonated water and dried in vacuum until the white powder was obtained, which was then redispersed in decarbonated water and used in the next step. Similar synthesis methods have been reported by different articles [27,40].

### 3.5. Characterization of Samples

*Negative-Staining (NS) TEM Observations.* NS-TEM images were recorded on a JEM-1011 TEM (JEOL, Japan) operating at an accelerating voltage of 100 kV. A drop of sample solution was loaded onto a 300-mesh carbon-coated copper grid and allowed to stand for 30 s. The excess solution was blotted off with filter paper, followed by staining with uranyl acetate solution (5 μL, 1% *w*/*v*). The specimens were kept in a desiccator overnight before observation.

*Dynamic light scattering (DLS) measurements.* DLS was carried out at a scattering angle of 90°. A standard laser light scattering spectrometer (Brookhaven, England) equipped with a coherent radiation 200 mW diode-pumped solid-state 488 nm laser and a Brookhaven Instruments Corporation (BI-9000AT) correlator was used for the measurements. The obtained data were analyzed by CONTIN.

*Steady-state fluorescence measurements.* The steady-state fluorescence measurements were performed on a fluorescence spectrophotometer (Hitachi F-7000, Japan) with a 150 W Xe lamp. The fluorescence emission spectra of pyrene probe (1.0 μM, Aladdin) in lipid solution were recorded between 350 nm and 550 nm by excitation at 335 nm, using excitation and emission slit widths of 5 and 2.5 nm, respectively. All measurements were taken at 25 ± 0.5 °C.

*Atomic Force Microscope (AFM) characterization.* A Nanoscope IIIa Multimode AFM (Digital Instruments Corp. USA) was used to examine the morphology of the aggregates in solutions deposited on mica wafers. AFM images were acquired in tapping mode using a Si tip cantilever with a force constant of 40 N∙m^−1^.

*Scanning electron microscope (SEM) characterization.* SEM images were recorded on a Gemini SEM 300 (Zeiss, Germany). Samples for SEM measurement (on a 300-mesh carbon-coated copper grid) were sputtered with a 5 nm thick platinum coating.

*Optical and confocal fluorescence microscopy.* Optical microscopy experiments were carried out on an Axioskop 40 optical microscope (Carl Zeiss, Germany). Fluorescence imaging was performed using an Olympus 1X81, and CE-LDHs were excited by using a filter with the following excitation (*λ*_ex_) and emission wavelength (*λ*_em_) cut-offs: *λ*_ex_ = 515–560 nm and *λ*_em_ = 580 nm. Confocal fluorescence microscopy measurements were performed using an Olympus IX81 fluorescence microscope (Olympus Optical Co., Ltd., Japan). A total of 10 μL of an aqueous solution of Nile red (*M_w_* = 318.38 g∙mol^−1^; 0.01 mg∙mL^−1^; TCI, Shanghai, China), calcein (*M_w_* = 622.53 g∙mol^−1^; 0.01 mg∙mL^−1^; Sigma, Shanghai, China) was added to 100 μL lipid vesicle solution (8 mM), where the optical excitations were carried out with 488 nm and 445 nm argon laser beam, and fluorescence emissions were detected over a range of 560–630 nm and 470–610 nm. The uptake or exclusion of the solutes was monitored by fluorescence microscopy.

*Zeta potential measurements.* All measurements were performed using a Malvern Zetasizer Nano-ZS instrument equipped with an internal Peltier temperature controller. The measurements were carried out in a disposable zeta cuvette at 25 °C.

*FT-IR and UV-vis measurements.* FT-IR spectra were recorded on a Vector 22 spectrometer (Bruker AXS Co., Ltd., Germany) in air at ambient temperature using the KBr disk method. UV-vis absorption spectra were recorded using a Shimadzu UV-1800 spectrometer (Shimadzu Inc., Kyoto, Japan). The absorbance was measured at 495 nm.

## 4. Conclusions

In a nutshell, the interaction of LDH NPs with model lipid membranes was investigated with the aid of different characterizations, including TEM, OM, FL, SEM, AFM, DLS, steady-state fluorescence measurements, etc. Microscopy results showed that the LDHs-SLB could first form around 1 h and, subsequently, LDH NPs could take around at least one more hour to be internalized in liposomes in the given concentration during the lipid fast exchange. A calcein leakage assay and steady-state fluorescence measurement were performed to check the membrane integrity and polarity of the pyrene-located microenvironment during the interaction between EYL and (CE)-LDHs, respectively. The oleophilic dye Nile red is dramatically encapsulated between the hydrophobic shell (*t* ≈ 2 h), while the negatively charged dye calcein is gradually taken up into the lumen (*t* ≈ 7 days). The so-obtained LDHs-100@EYL exhibits good stability during a storage period of two months at room temperature. However, precipitation occurs for LDHs-500/EYL dispersions. The realization of internalization of two-dimensional structured LDH NPs with different sizes into liposomes helps us to summarize the interactions between NPs and lipid membranes, which is not only an interesting route for creating interesting colloidal hybrids taking benefit of the merits of both LDHs with an average size ranging from 100 nm to 500 nm and liposomes, but also paving the way for LDHs to be used as drug delivery in vivo instead of in vitro experiments.

## Data Availability

The data presented in this study are available on request from the corresponding authors.

## Supplementary Materials

The following supporting information can be downloaded at https://www.mdpi.com/article/10.3390/molecules28093929/s1. Figure S1: Liposome size distribution histogram through analyzing OM images (a) without using extruder and (b) the cloudy solution extruded 11 times through two stacked polycarbonate membranes (pore size = 3 μm); (c,d) size distribution profiles of LDHs-100 and LDHs-500 particles from DLS measurement; Figure S2: (a) XRD pattern of LDHs-100 and (b) LDHs-500; Figure S3: (a) XRD pattern of LDHs-100 and (b) CE-LDHs-100. (c) SAXA pattern of CE-LDHs-100; Figure S4: FT-IR spectra of (a) calcein, (b) LDHs-100, and (c) CE-LDHs-100; Figure S5: (a) Variation of emission intensity of CE@EYL at 568 nm as a function of time and (b) variation of absorbance intensity of NR@EYL at 597 nm as a function of time; Figure S6: (a,b) HR-TEM image of liposomes when mixing with LDHs-500 in the volume ratio of 1/4 after standing 2 h. Scale bar: (a) 2 nm, (b) 200 nm; Figure S7: AFM images of (a) liposomes, (b) LDHs-500/EYL under the volume ratio of 1/4 after 2 h, and (c,d) AFM section analyses of (a,b); Table S1: XRD data of the diffraction peak; Table S2: Cell parameter of the sample.

## Sample Availability

Samples of the LDH NPs are available from the authors.
