# Supplementary material for: Interactions between Layered Double Hydroxide Nanoparticles and Egg Yolk Lecithin Liposome Membranes
Source: Molecules. 2023 May 6;28(9):3929. doi: 10.3390/molecules28093929 (PMC10180114; doi:10.3390/molecules28093929)
Supplement: Supplementary file 1 [file molecules-28-03929-s001.zip › molecules-2337987-supplementary.pdf]

Supporting Information

Interactions between Layered Double Hydroxide Nanoparticles and Egg Yolk Lecithin Liposome Membranes

Bin Liu [1,2], Yanlan Wang [1,*] and Na Du[2,*]

[1] School of Chemistry and Chemical Engineering, Liaocheng University, Liaocheng 252059, China; binliu@lcu.edu.cn

[2] Key Laboratory of Colloid and Interface Chemistry (Ministry of Education), School of Chemistry and Chemical Engineering, Shandong University, Jinan 250100, China

* Correspondence: wangyanlan@lcu.edu.cn (Y.L.W.); duna@sdu.edu.cn (N.D.)

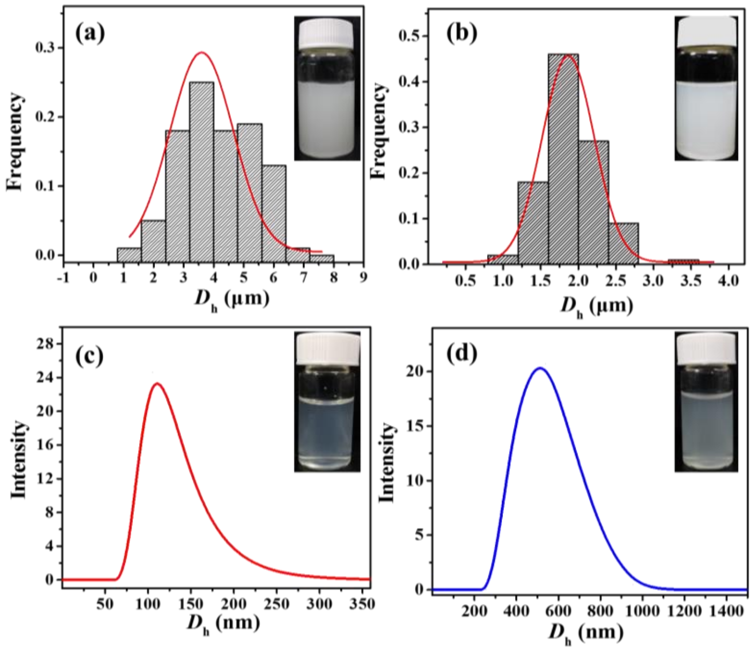

Figure S1. Liposome size distribution histogram through analyzing OM images (a) without using extruder and (b) the cloudy solution was extruded 11 times through two stacked polycarbonate membranes (pore size = 3 μm); (c, d) Size distribution profiles of LDHs-100 and LDHs-500 particles from DLS measurement.

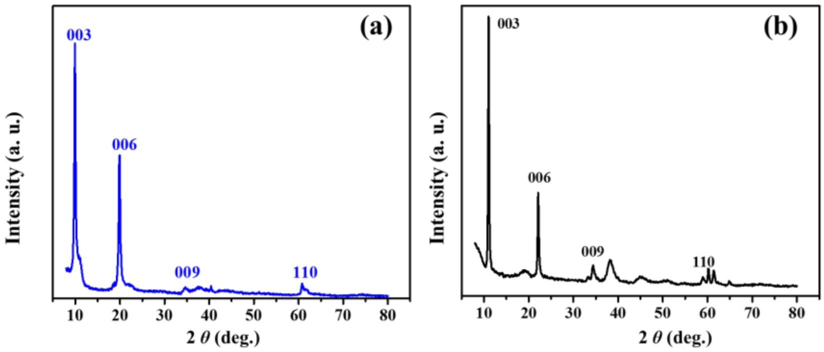

Figure S2. (a) XRD pattern of LDH-100 and (b) LDHs-500.

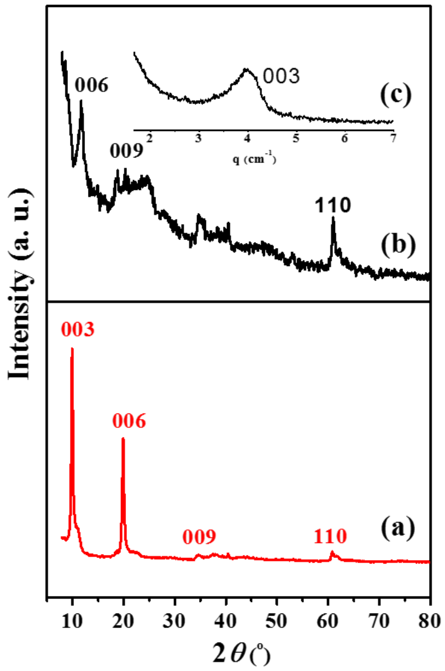

Figure S3. (a) XRD pattern of LDH-100 and (b) CE-LDHs-100. (c) SAXA pattern of CE-LDHs-100.

Table S1. XRD data of the diffraction peak.

Sample	003（2θ/°）	d/nm	006（2θ/°）	009（2θ/°）	110（2θ/°）
LDHs-100	9.92	0.89	19.92	34.80	61.60
LDH-500	11.06	0.79	22.16	34.40	60.16
CE-LDHs-100	5.50	1.60	11.65	18.89	61.01

Table S2. Cell parameter of the sample.

Sample	a=2d (110)/nm	c=3d (110)/nm
LDHs-100	0.30	2.67
LDH-500	0.31	2.37
CE-LDHs-100	0.30	4.80

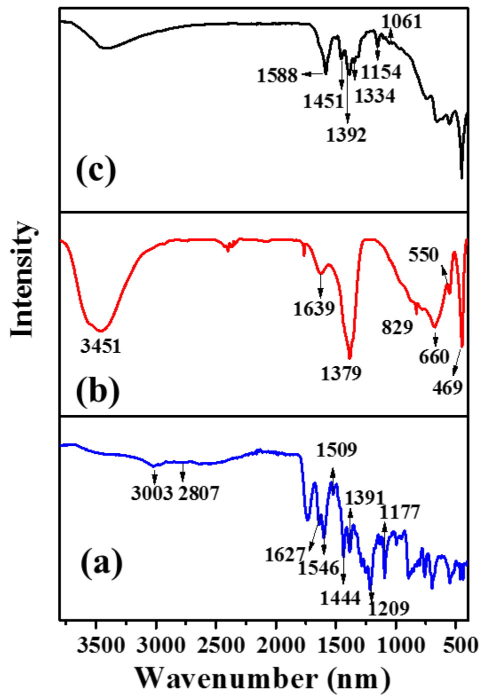

Figure S4. FT-IR spectra of (a) calcein, (b) LDHs-100 and (c) CE-LDHs-100.

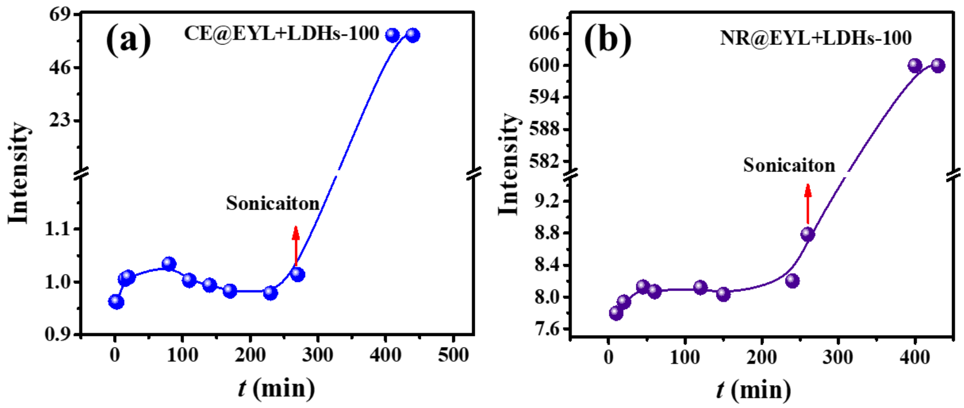

Figure S5. (a) Variation of emission intensity of CE@EYL at 568 nm as a function of time (b) Variation of absorbance intensity of NR@EYL at 597 nm as a function of time.

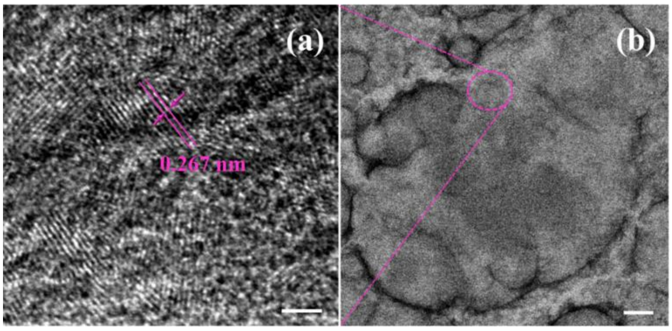

Figure S6. (a, b) HR-TEM image of liposomes when mixing with LDHs-500 in the volume ratio of 1/4 after standing 2 h. Scale bar: (a) 2 nm, (b) 200 nm.

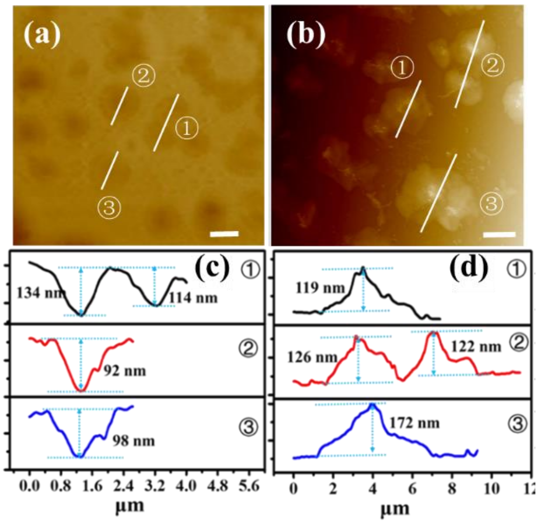

Figure S7. AFM images of (a) liposomes, (b) LDHs-500/EYL under the volume ratio of 1/4 after 2 h, (c, d) AFM section analyses of (a) and (b).
